# Ethnic disparities in stillbirth risk in Yunnan, China: a prospective cohort study, 2010-2018

**DOI:** 10.1186/s12889-020-10102-y

**Published:** 2021-01-14

**Authors:** Yanpeng Wu, Jianhong Pan, Dong Han, Lixin Li, Yanfei Wu, Rui Liao, Zijie Liu, Dingyun You, Pingyan Chen, Ying Wu

**Affiliations:** 1 State Key Laboratory of Organ Failure Research, Department of Biostatistics, Guangdong Provincial Key Laboratory of Tropical Disease Research, School of Public Health, Southern Medical University, Guangzhou, 510515 China; 2 The Third Affiliated Hospital of Southern Medical University, Guangzhou, 510515 China; 3 The First Affiliated Hospital of Zhengzhou University, Zhengzhou, 450052 China; 4 School of Public Health, Kunming Medical University, NHC Key Laboratory of Periconception Health Birth in Western China, Kunming, 650500 China; 5 The First Affiliated Hospital of Kunming Medical University, Kunming, 650500 China

**Keywords:** Stillbirth, Ethnic disparity, China

## Abstract

**Background:**

Racial and ethnic disparities in stillbirth risk had been documented in most western countries, but it remains unknown in China. This study was to determine whether exist ethnic disparities in stillbirth risk in mainland China.

**Methods:**

Pregnancy outcomes and ethnicity data were obtained from the National Free Preconception Health Examination Project (NEPHEP), a nationwide prospective population-based cohort study conducted in Yunnan China from 2010-2018. The Han majority and other four main minorities including Yi, Dai, Miao, Hani were investigated in the analysis. The stillbirth hazards were estimated by life-table analysis. The excess stillbirth risk (ESR) was computed for Chinese minorities using multivariable logistic regression.

**Results:**

Compared with other four minorities, women in Han majority were more likely to more educated, less multiparous, and less occupied in agriculture. The pattern of stillbirth hazard of Dai women across different gestation intervals were found to be different from other ethnic groups, especially in 20-23 weeks with 3.2 times higher than Han women. The ESR of the Dai, Hani, Miao, and Yi were 45.05, 18.70, -4.17 and 12.28%, respectively. Adjusted for maternal age, education, birth order and other general risk factors, the ethnic disparity still persisted between Dai women and Han women. Adjusted for preterm birth further (gestation age <37 weeks) can reduce 16.91% ESR of Dai women and made the disparity insignificant. Maternal diseases and congenital anomalies explained little for ethnic disparities.

**Conclusions:**

We identified the ethnic disparity in stillbirth risk between Dai women and Han women. General risk factors including sociodemographic factors and maternal diseases explained little. Considerable ethnic disparities can be attributed to preterm birth.

**Supplementary Information:**

The online version contains supplementary material available at 10.1186/s12889-020-10102-y.

## Background

Stillbirth, the death of a fetus in pregnancy before delivery, is one of the most common adverse pregnancy outcomes, accounting for two-thirds of perinatal deaths [1]. It is reported that about 2.6 million stillbirths occurred every year, 98% of which occurred in low- and middle-income countries [2]. The burden of stillbirth seems to be more serious in China [3–5]. Although the stillbirth rate in China has declined by 4-6% from 2000 to 2015 [6], the rate in 2016 still arrives at about 8.8 per 1000 births (95% CI 8.8-8.9) [7], which represents about 150,000 stillbirths annually in China.

Much attention has been paid to stillbirth and efforts have been made to identify causes [8–11]. Understanding the epidemiology of stillbirth in various populations is crucial to addressing this issue and it has motivated increasing research on racial/ethnical disparities in stillbirth risk in the United States, England, the Netherlands and other western countries, by which racial disparities have been identified among Blacks, Whites and Hispanics [12–16]. However, China, a typical multi-ethnic population country composed of Han and 55 other ethnic minorities, has few studies focused on the epidemiology of stillbirth among Chinese ethnic minorities [7]. It remains unclear whether stillbirth is more prevalent in ethnic minorities compared with the Han majority and what are the risk factors for the disparity, if any.

Like many other countries [13–15], ethnic minorities in China face similar disadvantaged social environments including living in remote areas far from cities, having lower levels of education, lower incomes, and lower utilization of medical care compared to the majority population [17, 18], all of which are known risk factors for adverse pregnancy outcomes including stillbirth [7, 12]. Other recognized or potential but unknown factors associated with stillbirth may also differ in distributions among different ethnic groups [19]. Thus, the ethnic disparity in stillbirth risk may be due to the fact that different levels of these factors are distributed in different proportions in different populations. Therefore, we investigated the risk of stillbirth in different ethnic groups in China to answer the question of whether and to what extent there are ethnic disparities in stillbirth risk in the Chinese population. Also, we sought to identify factors that contribute to ethnic disparities in stillbirth risk.

## Methods

### Geographic and demographic characteristics

The study was conducted in Yunnan province, which located in southwest China and bordered Myanmar in the west and Laos and Vietnam in the south. Yunnan is distinct from other provinces of China for a very high level of ethnic diversity and is the only province including all of China’s 55 minorities [20]. Over 38% of the province population are members of ethnic minorities, including the Yi, Bai, Hani, Dai, Miao, and so on.

### The NFPHEP project in Yunnan

The National Free Preconception Health Examination Project (NFPHEP) was a nationwide prospective population-based study, implemented by the Chinese National Health and Family Planning Commission and Ministry of Finance in 220 pilot counties in 30 provinces of China, to offer free preconception health examinations to rural married couples who planned to conceive within the next 6 months. General information including parental characteristics, medical and reproductive history, living habits and other exposing status related to adverse birth outcomes were recorded by local health workers. The pregnancy outcomes were identified by local hospitals. Detailed design and implementation of the NFPHEP are described elsewhere [21, 22].

This study was based on the data on couples enrolled in the NFPHEP from Yunnan province during 2010–18. By 27 August 2018, there were 1,140,417 families enrolled, of which 223,422 women conceived within 6 months were closely followed. Final records showed that 217,070 women had already delivered, 3,668 remained undelivered until the end of the study and 2,684 dropped out of the last follow-up. Our study focused on 217,070 gestations with definite outcomes, of which the top five ethnic groups accounted for 88.25%, where Han accounted for 61.34%, Yi 16.96%, Dai 4.47%, Miao 2.77% and Hani 2.71%. The detailed statistics of participated ethnic groups were displayed in Supplementary Table A, Additional Files.

### Definition and Assessment of variables

For stillbirth, we used the definition of fetal loss occurred on or after 20 weeks or birth body weight over 500 g if gestational age (GA) was unavailable [23, 24]. Fetus loss referred to those deaths prior to the complete expulsion or extraction from its mother, irrespective of the duration of pregnancy [1]. The gestation age was primarily determined by the interval between the first day of the last menstrual period and the date of delivery, and birth weight was measured within the first hour after delivery [1]. Ethnicity information was collected based on identification card of participants.

A variety of factors previously reported to be associated with racial or ethnic disparities had been incorporated in this paper with the following: maternal sociodemographic characteristics including maternal age, education, BMI, height, occupation (farmers and non-farmer), economic stress [8, 11, 15, 25–27]. Economic stress in our study was self-reported according to the question “how much stressed do you feel in your economic situation?” with the options “never”, “slightly”, “considerable” ; pregnancy-associated characteristics including parity, adverse pregnancy history (induced abortion, natural abortion and stillbirth) and birth order [8, 15], and maternal diseases (any report on maternal health reports including maternal hypertension, thyroid disease, syphilis, hepatitis B, anemia, diabetes, renal diseases and epilepsy) [8, 15]; maternal substance use before and during early pregnancy including tobacco, folate and Intrauterine devices (IUD) [11]; and fetal characteristics including fetal sex, gestation age and the diagnosis of congenital anomaly [8, 15]. Continuous variables were further categorized as follows based on a previous risk stratification. Maternal age was divided into 3 groups: <20 years, 20-35 years, >35 years [24, 26]. BMI (kg/m^2^) were grouped as underweight (<18.5), normal weight (18.5-24.9), overweight (25-29.9), obese (≥30) according to conventional World Health Organization (WHO) [27]. Maternal height was dichotomized as short maternal height (<150 cm) and non-short maternal height(≥150 cm) [8]. Education level was classified into 3 groups: low (completed primary school or lower), middle (completed middle school), high (completed high school or higher), to avoid categories with small number of participants. For categorical factors whose missing value proportion were over 20%, we treated their missing values as a separate category rather than discarded them directly in our analyses.

### Statistical analysis

We mainly incorporated the Han majority and other four main minorities including Yi, Dai, Miao, Hani into the analysis. Principal analysis was limited to singleton gestations that delivered during 20-42 weeks’ gestation, with the best clinical estimate of gestation age [28]. Pregnancies with missing plurality and gestation age would be firstly removed. The possible selection bias resulted from missing plurality and gestation age would be identified by the sensitivity analysis, which would be presented in the discussion section.

Comparisons concerning maternal and fetus characteristics among five ethnic groups were performed by chi-squared tests. The analyses to evaluate the influence of maternal age, education and birth order on stillbirth were stratified by ethnicity. Ethnic disparities were examined in the subsets of gestational age fell in 20-23 weeks, 24-27 weeks, 28-31 weeks, 32-36 weeks, 37-40 weeks, and 41-42 weeks, respectively. Stillbirth hazards were estimated by life-table analysis as the number of stillbirths occurring during different intervals divided by the number of ongoing pregnancies at the beginning of the corresponding intervals minus half of the total live births in this interval. The relative rate (RR) with 95% confidence intervals (95% CI) of stillbirth hazard (the Hans were the reference group) was calculated in each gestational age interval [29].

Multivariable logistic regressions were modeled by sequentially controlled several sets of covariates. In model 1, we examined the association between maternal ethnicity and stillbirth risk after maternal age, education level, and birth order controlled which had been considered as important confounders by prior researches [7, 12, 25]. In model 2, we added smoking status, BMI, height, occupation, economic stress, folate use and IUD use, adverse pregnancy history. In model 3, we introduced the preterm birth variable (gestation age <37 weeks) known to be an important mediator of risk of stillbirth [12]. Compared with the Han majority, the excess stillbirth risk (ESR) in minorities (%) were computed as follows:

$$ \mathrm{Excess}\ \mathrm{stillbirth}\ \mathrm{risk}=\frac{\left( RR-1\right)}{RR}, $$

where RR = adjusted relative risk of stillbirth. The adjusted odds ratios were used to approximate the RR. We used the ESR to reflect the magnitude of ethnic disparities in stillbirth risk. The analysis was repeated again in the group of women excluding those with maternal diseases and the group of deliveries excluding those congenital anomalies, respectively. All analysis was performed by R statistical software (R.3.6).

## Results

We incorporated 191,560 pregnancies from five selected ethnic groups. We then sequentially excluded 11,419 pregnancies with missing plurality and gestational age, 965 multiple births, 1,644 births with gestational age at delivery <20 and >42 weeks’ gestation, 11 induced abortions whose gestation age beyond 20 weeks with unknown reasons were also removed. After the above exclusions, 177,520 singleton births remained for analysis including 176,434 live births and 1,086 stillbirths (0.62%). The geographical distribution of included births showed obvious ethnic aggregations in Yunnan province (Supplementary Figure A, Additional Files).

These five ethnic groups differed significantly among maternal and fetus characteristics (Table 1). As a majority, Han women were more likely to more educated, less multiparous, and less occupied in agriculture. For other minorities, Miao women have a younger childbearing age and a lower education level. Hani women underwent more economic stress. Dai women were more frequent in multiparous and IUD use. Notably, Dai women had the highest prevalence in stillbirth (1%) and preterm birth (7.6%).

**Table 1:** The distribution of maternal and fetus characteristics among five ethnic groups: Yunnan, China, 2010-2018

	Dai	Han	Hani	Miao	Yi	Total	***P*** Value
**Number**	8,733	123,600	5,292	5,639	34,256	177,520	
**Sociodemographic characteristics, No. (%)**							
**Education level**							< 0.001
N-Miss	20	1,591	64	30	246	1,951	
Low	2,767 (31.8)	1,7696 (14.5)	1,733 (33.1)	2,990 (53.3)	9,255 (27.2)	34,441 (19.6)	
Middle	4,636 (53.2)	6,9643 (57.1)	2,514 (48.1)	2,203 (39.3)	18,391 (54.1)	97,387 (55.5)	
High	1,310 (15.0)	3,4670 (28.4)	981 (18.8)	416 (7.4)	6,364 (18.7)	43,741 (24.9)	
**Parity**							< 0.001
N-Miss	17	375	21	11	90	514	
0	2,553 (29.3)	51,873 (42.1)	1,495 (28.4)	1,990 (35.4)	11,751 (34.4)	69,662 (39.4)	
1	3,752 (43.0)	48,116 (39.0)	1,896 (36.0)	2,297 (40.8)	13,417 (39.3)	69,478 (39.3)	
>1	2,411 (27.7)	23,236 (18.9)	1,880 (35.7)	1,341 (23.8)	8,998 (26.3)	37,866 (21.4)	
**Age**							< 0.001
N-Miss	26	146	11	32	25	240	
<20	334 (3.8)	3,236 (2.6)	231 (4.4)	880 (15.7)	1,134 (3.3)	5,815 (3.3)	
20-35	7,979 (91.6)	112,455 (91.1)	4,663 (88.3)	4,393 (78.3)	31,259 (91.3)	160,749 (90.7)	
≥35	394 (4.5)	7,763 (6.3)	387 (7.3)	334 (6.0)	1,838 (5.4)	10,716 (6.0)	
**Birth order**							< 0.001
N-miss	17	375	21	11	90	514	
1	3,681 (42.2)	62,506 (50.7)	2,176 (41.3)	2,307 (41.0)	15,509 (45.4)	86,179 (48.7)	
2	4,901 (56.2)	59,077 (47.9)	2,897 (55.0)	3,074 (54.6)	18,080 (52.9)	88,029 (49.7)	
>3	134 (1.5)	1,642 (1.3)	198 (3.8)	247 (4.4)	577 (1.7)	2,798 (1.6)	
**Work**							< 0.001
N-Miss	37	1,226	58	63	227	1,611	
Farmer	8,390 (96.5)	111,618 (91.2)	4,965 (94.9)	5,459 (97.9)	32,605 (95.8)	163,037 (92.7)	
Non-farmer	306 (3.5)	10,756 (8.8)	269 (5.1)	117 (2.1)	1,424 (4.2)	12,872 (7.3)	
**BMI (kg/m**^**2**^**)**							< 0.001
N-Miss	3	232	6	4	38	283	
Underweight	1,553 (17.8)	16,969 (13.8)	632 (12.0)	429 (7.6)	4,174 (12.2)	23,757 (13.4)	
Normal weight	6,145 (70.4)	93,793 (76.0)	3,946 (74.7)	4,678 (83.0)	26,382 (77.1)	134,944 (76.1)	
Overweight	877 (10.0)	11,179 (9.1)	597 (11.3)	471 (8.4)	3,176 (9.3)	16,300 (9.2)	
Obese	155(1.8)	1,427 (1.2)	111 (2.1)	57 (1.0)	486 (1.4)	2,236 (1.3)	
**Height (cm)**							
N-miss	3	232	6	4	38	283	
<150	1,397 (16.0)	10,827 (8.8)	917 (17.3)	1,998 (35.5)	4,181 (12.2)	19,320 (10.9)	
≥150	7,333 (84.0)	112,541 (91.2)	4,369 (82.7)	3,637 (64.5)	30,037 (87.8)	157,917 (89.1)	
**Maternal disease** ^**a**^							< 0.001
N-Miss	71	981	64	31	286	1,433	
Yes	261 (3.0)	1,579 (1.3)	101 (1.9)	42 (0.7)	388 (1.1)	2,371 (1.3)	
**Economic stress**							< 0.001
N-Miss	51	700	39	31	187	1,008	
Never	6,705 (77.2)	91,354 (74.3)	3,249 (61.9)	4,546 (81.1)	24,572 (72.1)	130,426 (73.9)	
Slightly	1,157 (13.3)	18,704 (15.2)	1,023 (19.5)	606 (10.8)	5,317 (15.6)	26,807 (15.2)	
Considerable	820 (9.4)	12,842 (10.4)	981 (18.7)	456 (8.1)	4,180 (12.3)	19,279 (10.9)	
**Past obstetric history, No. (%)**							
**Induced abortion**							< 0.001
Yes	2,845 (32.6)	26,052 (21.1)	1,998 (37.8)	1,239 (22.0)	10,137 (29.6)	42,271 (23.8)	
Missing	2,537 (29.1)	45,518 (36.8)	1,433 (27.1)	1,971 (35.0)	10,998 (32.1)	62,457 (35.2)	
**Natural abortion**							< 0.001
Yes	356 (4.1)	4,139 (3.3)	302 (5.7)	195 (3.5)	1,298 (3.8)	6,290 (3.5)	
Missing	2,537 (29.1)	45,520 (36.8)	1,433 (27.1)	1,971 (35.0)	10,998 (32.1)	62,459 (35.2)	
**Stillbirth**							< 0.001
Yes	68 (0.8)	1,024 (0.8)	60 (1.1)	43 (0.8)	309 (0.9)	1,504 (0.8)	
Missing	2,537 (29.1)	45,520 (36.8)	1,433 (27.1)	1,971 (35.0)	10,998 (32.1)	62,459 (35.2)	
**Maternal substance use, No. (%)**							
**Folate use**							< 0.001
N-Miss	14	784	31	51	192	1,072	
Unused	420 (4.8)	5,092 (4.1)	464 (8.8)	351 (6.3)	1591 (4.7)	7,918 (4.5)	
Irregular Used	475 (5.5)	7,831 (6.4)	411 (7.8)	355 (6.4)	3,092 (9.1)	12,164 (6.9)	
Regular Used	7,824 (89.7)	109,893 (89.5)	4,386 (83.4)	4,882 (87.3)	29,381 (86.3)	156,366 (88.6)	
**Smoking status**							< 0.001
N-Miss	69	953	48	39	247	1,356	
Smoke free	7,509 (86.7)	103,543 (84.4)	4,359 (83.1)	5,119 (91.4)	29,364 (86.3)	149,894 (85.1)	
Passive smoking (only)	1,130 (13.0)	18,635 (15.2)	856 (16.3)	473 (8.4)	4,570 (13.4)	25,664 (14.6)	
Smoker	25 (0.3)	469 (0.4)	29 (0.6)	8 (0.1)	75 (0.2)	606 (0.3)	
**IUD use**							< 0.001
N-Miss	38	822	31	34	185	1,110	
Ever	2,450 (28.2)	26,417 (21.5)	1,164 (22.1)	1,010 (18.0)	8,161 (24.0)	39,202 (22.2)	
**Current pregnancy outcomes, No. (%)**							
**Fetus sex**							0.007
N-miss	23	259	7	9	87	385	
Female	4,216 (48.4)	58,905 (47.8)	2,464 (46.6)	2,596 (46.1)	16,469 (48.2)	84,650 (47.8)	
Male	4,463 (51.2)	63,924 (51.8)	2,807 (53.1)	3,002 (53.3)	17,577 (51.4)	91,773 (51.8)	
Unknown ^b^	31 (0.4)	512 (0.4)	14 (0.3)	32 (0.6)	123 (0.4)	712 (0.4)	
**Preterm births** ^**c**^	667 (7.6)	6,235 (5.0)	351 (6.6)	364 (6.5)	1,607 (4.7)	9,224 (5.2)	< 0.001
**Congenital anomalies**							0.454
N-Miss	23	261	7	9	87	387	
Yes	13 (0.1)	159 (0.1)	7 (0.1)	5 (0.1)	31 (0.1)	215 (0.1)	
**Stillbirth**	90 (1.0)	705 (0.6)	37 (0.7)	31 (0.5)	223 (0.7)	1,086 (0.6)	< 0.001
Female stillbirth	25 (0.3)	218 (0.2)	13 (0.2)	12 (0.2)	67 (0.2)	335 (0.2)	
Male stillbirth	33 (0.4)	261 (0.2)	13 (0.2)	9 (0.2)	90 (0.3)	406 (0.2)	
Sex-unknown ^b^ stillbirth	31 (0.4)	224 (0.2)	11 (0.2)	10 (0.2)	64 (0.2)	340 (0.2)	
Sex-missing stillbirth	1 (0.0)	2 (0.0)	0 (0.0)	0 (0.0)	2 (0.0)	5 (0.0)	

*Note.* ‘N-Miss’ refer to the number of missing data. ‘Missing’ refer to missing value in categorical variables coded as a separate category.

^a^ Maternal disease including hypertension, thyroid disease, syphilis and hepatitis B

^b^ hermaphroditism or difficult to judge due to some birth defects.

^c^ Gestation age <37 weeks

**Fig. 1:**
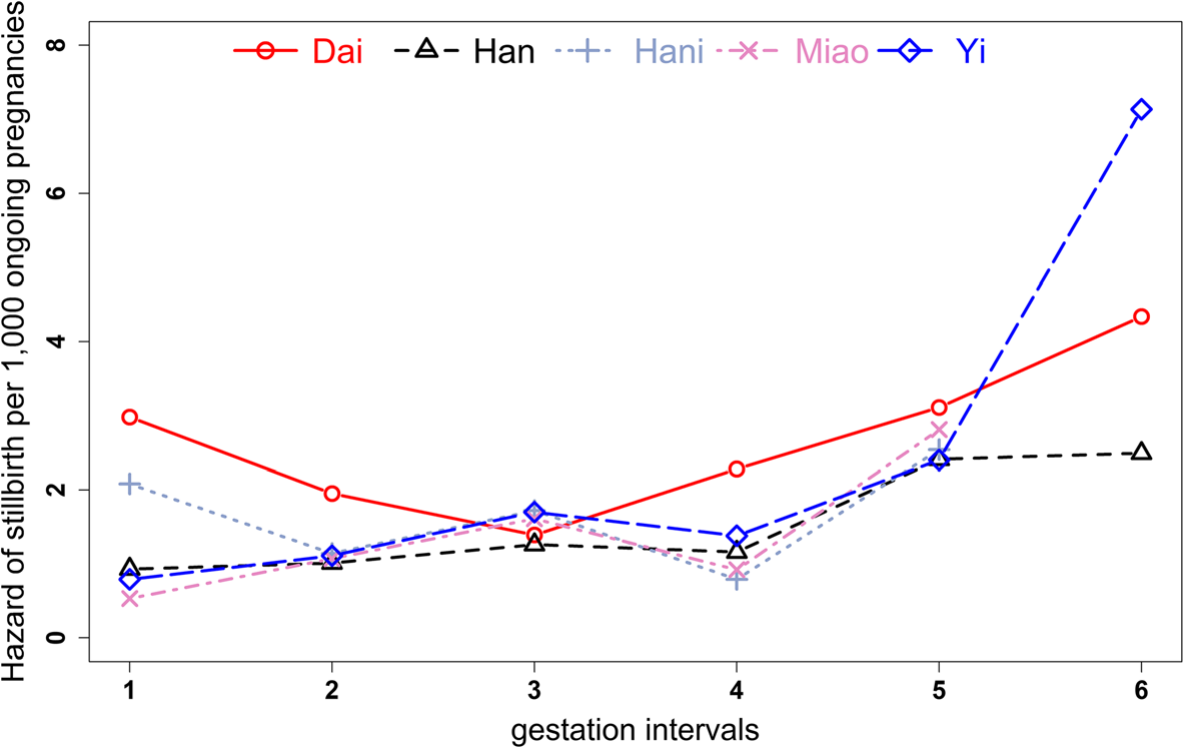
Hazard of stillbirth in different gestational intervals and maternal ethnicities (gestation intervals: 1, 20-23 weeks; 2, 24-27 weeks; 3, 28-31 weeks; 4, 32-36 weeks; 5, 37-41 weeks; 6, 41-42 weeks): Yunnan, China, 2010-2018

It found that the influence of maternal age, education and birth order on stillbirth varied by ethnicity (Table 2). Compared to other ethnic groups, Yi women were more likely influenced by age (RR 3.08, 95%CI 1.18-8.02) , education (RR 1.57, 95%CI 1.06-2.33) and birth order (RR 3.97, 95%CI 2.23-7.07). Figure 1 depicted stillbirth hazards of five ethnic groups in different gestational intervals. Han women, Yi women, Miao women showed a similar pattern of increased stillbirth hazard across 20-37 weeks. The significant ethnic disparities occurred in 20-23 weeks and 41-42 weeks. As Table 3 showed, the Dai/Han disparity in stillbirth hazard was highest at 20-23 weeks (RR 3.20, 95%CI 2.23-4.61). Hani/Han disparity occurred at 20-23 weeks (RR 2.24, 95% CI 1.33-3.77) and Yi/Han disparities at 41-42 weeks (RR 2.87, 95% CI 1.34-6.15).

**Table 2:** The ethnicity-specific stillbirth per 1000 pregnancies: Yunnan, China, 2010-2018

	Maternal Age	RR (95% CI)		Education	RR (95% CI)		Birth Order	RR (95% CI)	
	<20	20-35 vs <20	>35 vs <20	High	Middle vs High	Low vs High	1	2 vs 1	>2 vs 1
Dai	11.98	0.84 (0.31,2.28)	1.27 (0.36,4.46)	9.16	1.11 (0.59,2.09)	1.18 (0.61,2.30)	8.15	1.45 (0.94,2.25)	0.92 (0.13,6.70)
Han	5.56	0.97 (0.61,1.55)	1.76 (1.05,2.94)	5.77	0.9 (0.76,1.07)	1.28 (1.03,1.59)	4.70	1.39 (1.19,1.62)	2.85 (1.85,4.38)
Hani	4.33	1.73 (0.24,12.57)	0.60 (0.04,9.55)	5.10	1.48 (0.55,3.95)	1.47 (0.53,4.11)	3.22	3 (1.31,6.86)	3.14 (0.66,15.01)
Miao	5.68	0.96 (0.37,2.51)	1.05 (0.20,5.39)	4.81	1.04 (0.23,4.68)	1.25 (0.29,5.37)	4.33	1.35 (0.62,2.92)	2.8 (0.78,10.11)
Yi	4.41	1.40 (0.58,3.40)	3.08 (1.18,8.02)	5.50	1.03 (0.70,1.51)	1.57 (1.06,2.33)	5.67	1.19 (0.91,1.56)	3.97 (2.23,7.07)

*Note*. *CI* Confidence interval, *RR* Relative risk

**Table 3:** Relative rate of stillbirth hazard in different gestational age intervals: Yunnan, China, 2010-2018

Gestational age (weeks)	Number of deliveries (stillbirths)	Hazard/1000 ongoing pregnancies	Relative rate of stillbirth hazard to Han women (95% CI)			
		Han	Dai	Hani	Miao	Yi
20-23	234 (182)	0.93	3.20 (2.23,4.61)	2.24 (1.33,3.77)	0.57 (0.19,1.69)	0.85 (0.57,1.26)
24-27	357 (192)	1.01	1.93 (1.22,3.05)	1.13 (0.53,2.39)	1.06 (0.51,2.21)	1.10 (0.79,1.53)
28-31	1,150 (243)	1.26	1.10 (0.64,1.90)	1.37 (0.76,2.45)	1.28 (0.72,2.25)	1.35 (1.04,1.75)
32-36	7,483 (213)	1.16	1.97 (1.28,3.01)	0.68 (0.27,1.74)	0.79 (0.35,1.79)	1.19 (0.88,1.60)
37-40	151,291 (228)	2.41	1.29 (0.78,2.13)	1.05 (0.53,2.08)	1.17 (0.63,2.15)	1.00 (0.74,1.35)
41-42	17,005 (28)	2.49	1.74 (0.41,7.50)	0 ^a^	0 ^a^	2.87 (1.34,6.15)

*Note.* Han women were the reference group

*CI* Confidence interval

^a^ No stillbirth in that gestational interval

**Fig. 2:**
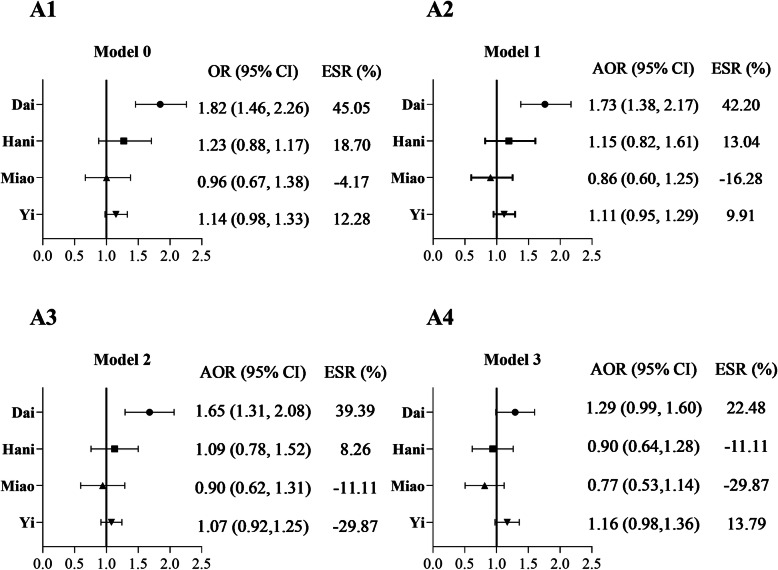
Adjusted odds ratios for stillbirth of ethnic minorities: Yunnan, China, 2010-2018. *Note.* OR: Odds ratio (Han women are the reference group). AOR: Adjusted odds ratio. ESR: Excess stillbirth risk, i.e. [(OR-1)/OR] ×100%, where OR approximates the adjusted relative risk (Han women are the reference group). Panel A1: Model 0, unadjusted OR for stillbirth risk. Panel A2: Model 1, adjusted for the effects of maternal age, birth order, education level. Panel A3: Model 2, adjusted for the effects of maternal age, birth order, education level, smoking, BMI, height, occupation, economic stress, IUD use, folate use, adverse pregnancy history. Panel A4: Model 3, adjusted for all the above plus preterm birth

Crude and adjusted odds ratios (OR) for singletons stillbirth were presented in Figure 2. The ESR of Dai women was 42.20% after maternal age, birth order, and education were controlled (Figure 2, Panel A2). After other factors including smoking, BMI, height, occupation, economic stress, folate use, IUD use, adverse pregnancy history were further introduced, the ESR of Dai women changed little (Figure 2, Panel A3). However, after the addition of preterm birth, the ESR of Dai women reduced to 22.48% from 39.39% and lost statistical significance (Figure 2, Panel A4). Similar patterns were observed in the group of women without maternal diseases and the group of pregnancies excluded congenital anomalies, respectively (Supplementary Tables B and C, Additional Files).

## Discussion

### Main findings

Using data from 177,520 singleton pregnancies from five ethnic groups in Yunnan during 2010-2018, we initially found ethnic disparities in stillbirth risk, where Dai women had a higher stillbirth rate (1%) than other four ethnic groups. The pattern of stillbirth hazard of Dai women across gestation was found to be different from other ethnic groups, especially in 20-23 weeks with 3.2 times higher than Han women. The disparity in stillbirth risk between Dai women and Han women still persisted even adjusted for maternal age, education level, birth order and other general risk factors. The control of the effect of preterm birth was found to reduce 16.91% ESR of Dai women and make Dai/Han disparity insignificant.

### Comparison with the existing literature

To date, there was little literature reported disparities in stillbirth risk of Chinese ethnic groups. Three of the largest cohort studies concerning stillbirth in China all failed to introduce maternal ethnicity into analysis [6, 7, 11]. Most of the researches concerning Chinese ethnic disparities focused on maternal and child mortality [30]. Our study was the first study to target ethnic disparities in stillbirth. Compared with foreign studies concerning racial/ethnic disparity, it was consistent that minorities had a higher stillbirth risk compared with the major ethnic group [15, 25]. Moreover, preterm birth was found to be an important factor for increased risk of stillbirth in Dai women, while a similar finding was also revealed in Non-Hispanic black women in the United States [12, 25].

### Interpretations

As Table 3 and Figure 1 showed, preterm births contributed to a considerable proportion of stillbirths and the Dai/Han disparity disappeared after preterm birth was controlled. As known, preterm birth can result from spontaneous preterm labor, premature preterm rupture of membranes and medically indicated delivery [31]. Spontaneous preterm birth has been reported to cause substantial proportions of stillbirths especially intrapartum stillbirths at pre- and peri-viable gestation ages [12].

The low rate of maternal medical conditions of Dai women implied that their preterm births may be more likely to be spontaneous preterm births and that more efforts should be focused on this to reduce the ethnic disparity in stillbirth. On the other hand, we made a speculation that the higher rates of preterm birth and stillbirth among Dai women compared to other ethnic groups may be due to their own unique characteristics including history, customs, and habits. For example, living in a subtropical climate, eating an insect-eating diet, and preferring Dai traditional medicine that lacks scientific validation are potential risk factors that conspire to increase the risk of adverse pregnancy outcomes such as preterm delivery and stillbirth [32–35]. However, the data collected in our current study are not sufficient to support this speculation, and we look forward to further research on this issue in the future.

It should be also noted that deliveries by Yi women was found to be more affected by maternal age, education level and birth order (Table 2) relative to other ethnic groups, which could be considered as important area of focus for efforts to reduce stillbirth risk of Yi. It also showed that a significantly higher stillbirth risk in late gestation (41-42weeks) of Yi women (Fig. 1, Table 3). This may be attributed to living in rural mountains [30] and were less likely to get access to health care, which would lead to fewer antenatal visits and therefore late detections and bad prognoses.

In addition, birth order was included in this study, which was less studied in other nations. Compared to parity, birth order implied certain other meanings impacted by the one-child policy of China [36]. For example, the birth of first child tend to have more attention of families than the second child or child of the higher birth order. As shown in Table 2, there was an increasing risk of stillbirth with increasing birth order, which was consistent with previous studies in China [7]. The impact of the one-child policy on the current pregnancies may also act through adverse pregnancy history. As Table 1 shows, the proportion of ever having induced abortions and missing values accounted for almost two thirds totally. Especially, Han ethnicity was allowed to have only one child while most of minorities were relaxed to two children. Hence Han women with advanced age were more likely to have adverse pregnancy history. Yet one-child policy showed limited impact on the current pregnancies of this study compared before because of the one-child policy relaxation at late stages and fewer probabilities for participants who ready for conceive to have induced abortions caused by the policy [30].

As the project has been conducted and followed for 8 years, it was possible that some women had multiple pregnancies and were repeatedly included. Due to privacy of participants, we cannot identify information of those repeated participants. It’s intuitive that the women with a higher risk of stillbirth due to prior stillbirth histories were more likely to repeat participations during 8 years periods. Although the adverse pregnancy history was controlled, the independence assumption was actually violated and may lead to inconclusive results.

We also conducted sensitivity analyses to identify potential selection bias induced by excluding those pregnancies with missing values on plurality and gestation age. Firstly, the outcomes of 11,419 pregnancies with missing plurality and gestation age were identified from the pregnancy outcome records. Among these excluded pregnancies, there were 3,475 induced abortions, 6,820 spontaneous abortions, and 1,124 live births. The induced abortions and spontaneous abortions were beyond the scope of the research subjects. Thus, 1,124 live births who was not satisfy the inclusion criteria due to missing plurality and gestation age were added to analysis dataset to re-perform the multivariable analysis. According to the results, there was no substantial inconsistency across different results (Supplementary Table D, Additional Files). A second opportunity for selection bias arose if pregnancies became lost to follow-up. Since this project was an official agency-sponsored study, participants were more compliant and had a lower percentage of missing final outcomes (2,684 missing outcomes in 223,422 participants). Therefore, the extent to which the second selection bias affects the results should be small.”

### Limitations

Our study failed to capture more information associated with prenatal care which might play important roles in explaining the ethnic disparity found [30]. Although maternal education and the folate use, which can be regarded as correlated factors to prenatal care, had been used to remove the mediated effect of prenatal care, there were still other important but uncollected factors like prenatal visits which may explain the remain ethnic disparities. Short birth interval is also an important a risk factor to preterm birth and stillbirth [12, 31] and may to some extent explain ethnic disparity in stillbirth risk, which, however, is not collected in this project and thus need more concerns in future research.

Also, the rate of congenital abnormalities in this study was lower (12/10,000) than reported intervals of about 101.74/10,000 to 140.85/10,000 in other studies [37]. One possible reason was that people participated in this project may pay more attention on health care by which possible congenital abnormality was timely detected by anatomy scan and reduced by surgical abortion. Another possible reason was that women who agreed to this project may be more likely to have a planned pregnancy than those who did not, and the latter may contain more unplanned pregnancies with poor prenatal care, which was a factor of adverse pregnancy outcomes. In this regard, our study focusing on enrolled population may underestimate stillbirth risk of the whole population in Yunnan.

### Implications

General risk factors including sociodemographic factors and maternal diseases may only explain little on ethnic disparities according to the association findings. We call for more measures and collections about biologic factors, prenatal care information, ethnic customs on minorities in future studies. Additionally, we should give more concern on the early pregnancy health of Dai women to reduce the possibility of stillbirth due to preterm birth. Identifying the ethnic disparities in stillbirth risk is meaningful for both health resource allocation and medical decisions, making from a public health perspective.

## Supplementary Information

**Additional file 1: Supplementary Table A.** The distribution of all pregnancy outcomes according to maternal ethnicity: Yunnan, China, 2010-2018. **Supplementary Figure A.** The geographic distribution of deliveries from the Han, Yi, Hani, Di, Miao: Yunnan, China, 2010-2018. **Supplementary Table B.** Adjusted odds ratios for stillbirth of ethnic minorities excluding women with medical disease: Yunnan, China, 2010-2018. **Supplementary Table C.** Adjusted odds ratios for stillbirth of ethnic minorities excluding deliveries with congenital anomalies: Yunnan, China, 2010-2018. **Supplementary Table D.** Adjusted odds ratios for stillbirth of ethnic minorities including deliveries with missing plurality and gestation age: Yunnan, China, 2010-2018

## Data Availability

The datasets generated and/or analyzed during the current study are not publicly available due to the privacy of the participants, but are available from the corresponding author on reasonable request.

## Abbreviations

BMI: Body Mass Index
CI: Confidence interval
ESR: Excess stillbirth risk
GA: Gestational age
IUD: Intrauterine devices
NFPHEP: National Free Preconception Health Examination Project
PTB: Preterm birth
RR: Relative rate
