# Supplementary material for: Ethnic disparities in stillbirth risk in Yunnan, China: a prospective cohort study, 2010-2018
Source: BMC Public Health. 2021 Jan 14;21:136. doi: 10.1186/s12889-020-10102-y (PMC7807874; doi:10.1186/s12889-020-10102-y)
Supplement: Supplementary file 1 — Additional file 1: Supplementary Table A. The distribution of all pregnancy outcomes according to maternal ethnicity: Yunnan, China, 2010-2018. Supplementary Figure A. The geographic distribution of deliveries from the Han, Yi, Hani, Di, Miao: Yunnan, China, 2010-2018. Supplementary Table B. Adjusted odds ratios for stillbirth of ethnic minorities excluding women with medical disease: Yunnan, China, 2010-2018. Supplementary Table C. Adjusted odds ratios for stillbirth of ethnic minorities excluding deliveries with congenital anomalies: Yunnan, China, 2010-2018. Supplementary Table D. Adjusted odds ratios for stillbirth of ethnic minorities including deliveries with missing plurality and gestation age: Yunnan, China, 2010-2018 [file 12889_2020_10102_MOESM1_ESM.docx]

**Supplement**

Supplementary Table A- The distribution of all pregnancy outcomes according to maternal ethnicity: Yunnan, China, 2010-2018

| **Race** | **Number of cases** | **Percentage** | **Number**  **of stillbirths** | | **Stillbirth**  **per 1000**  **pregnancies** |
| --- | --- | --- | --- | --- | --- |
| Han | 133,140 | 61.335 | | 740 | 5.56 |
| Yi | 36,810 | 16.958 | | 240 | 6.52 |
| Dai | 9,711 | 4.474 | | 93 | 9.58 |
| Miao | 6,008 | 2.768 | | 34 | 5.66 |
| Hani | 5,891 | 2.714 | | 42 | 7.13 |
| Bai | 4,528 | 2.086 | | 29 | 6.4 |
| Zhuang | 4,177 | 1.924 | | 38 | 9.1 |
| Lisu | 3,964 | 1.826 | | 36 | 9.08 |
| Hui | 2,098 | 0.967 | | 12 | 5.72 |
| Jingpo | 1,878 | 0.865 | | 16 | 8.52 |
| Lahu | 1,724 | 0.794 | | 17 | 9.86 |
| Wa | 1,708 | 0.787 | | 13 | 7.61 |
| Yao | 824 | 0.380 | | 9 | 10.92 |
| Tibetans | 654 | 0.301 | | 0 | 0 |
| Naxi | 650 | 0.299 | | 5 | 7.69 |
| Derung | 458 | 0.211 | | 6 | 13.1 |
| Achang | 413 | 0.190 | | 2 | 4.84 |
| Blang | 412 | 0.190 | | 1 | 2.43 |
| Mongols | 235 | 0.108 | | 0 | 0 |
| Buyi | 221 | 0.102 | | 2 | 9.05 |
| PUmi | 221 | 0.102 | | 0 | 0 |
| Nu | 63 | 0.029 | | 1 | 15.87 |
| Tujia | 57 | 0.026 | | 0 | 0 |
| Jino | 53 | 0.024 | | 0 | 0 |
| Li | 47 | 0.022 | | 1 | 21.28 |
| Man | 40 | 0.018 | | 0 | 0 |
| Dong | 23 | 0.011 | | 0 | 0 |
| Dulong | 14 | 0.006 | | 0 | 0 |
| Kelao | 12 | 0.006 | | 0 | 0 |
| Shui | 12 | 0.006 | | 0 | 0 |
| Tu | 12 | 0.006 | | 0 | 0 |
| Jing | 5 | 0.002 | | 0 | 0 |
| kazakh | 5 | 0.002 | | 0 | 0 |
| Koreans | 5 | 0.002 | | 0 | 0 |
| Gaoshan | 3 | 0.001 | | 0 | 0 |
| CQing | 2 | 0.001 | | 0 | 0 |
| Dongxiang | 2 | 0.001 | | 0 | 0 |
| Uyghur | 2 | 0.001 | | 0 | 0 |
| Dahaner | 1 | 0.000 | | 0 | 0 |
| Mosuo | 1 | 0.000 | | 0 | 0 |
| Qiang | 1 | 0.000 | | 0 | 0 |
| She | 1 | 0.000 | | 0 | 0 |
| Unknow* | 984 | 0.453 | | 4 | 4.07 |

*The ethnicity identify was missing


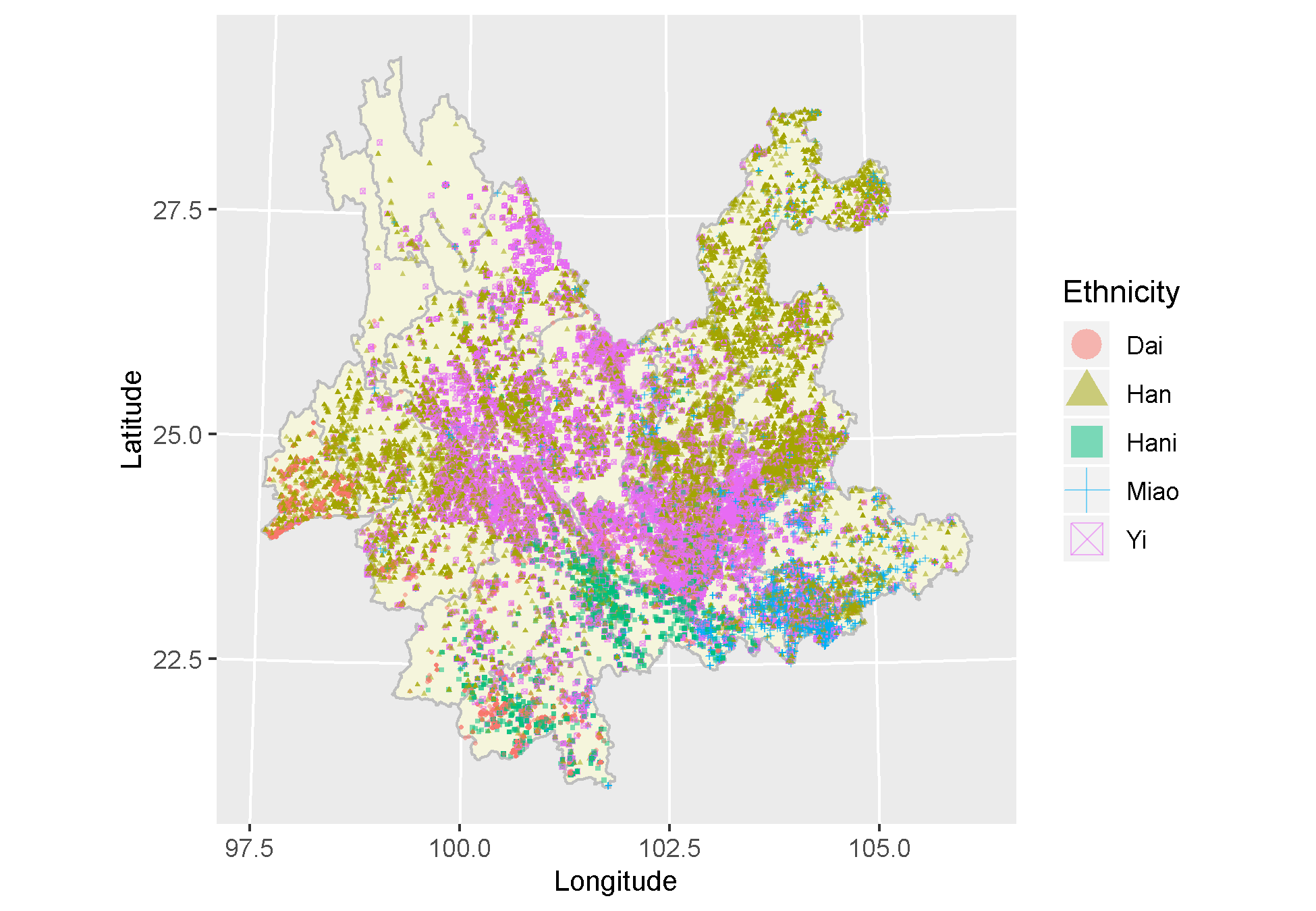


Supplementary Figure A. The geographic distribution of deliveries from the Han, Yi, Hani, Di, Miao: Yunnan, China, 2010-2018

Supplementary Table B-Adjusted odds ratios for stillbirth of ethnic minorities excluding women with medical disease: Yunnan, China, 2010-2018

|  | Crude  OR | ESR  (%) | Model 1^a^ | | Model 2^b^ | | Model 3^c^ | |
| --- | --- | --- | --- | --- | --- | --- | --- | --- |
|  |  |  | Adjusted OR | ESR  (%) | Adjusted OR | ESR  (%) | Adjusted OR | ESR  (%) |
| Dai | 1.77 (1.37, 2.18) | 43.50 | 1.68 (1.30, 2.09) | 40.48 | 1.61 (1.27, 2.04) | 37.89 | 1.25 (0.98, 1.60) | 20.00 |
| Hani | 1.21 (0.86, 1.70) | 17.36 | 1.14 (0.81, 1.60) | 12.28 | 1.07 (0.76, 1.52) | 6.54 | 0.89 (0.62, 1.27) | -12.36 |
| Miao | 0.95 (0.67, 1.38) | -5.26 | 0.86 (0.60, 1.26) | -16.28 | 0.91 (0.62, 1.32) | -9.89 | 0.78 (0.53, 1.15) | -28.21 |
| Yi | 1.16 (0.99, 1.35) | 13.79 | 1.12 (0.96, 1.31) | 10.71 | 1.08 (0.92, 1.27) | 7.41 | 1.16 (0.98, 1.37) | 13.79 |

*Note.* OR: Odds ratio (Han women are the reference group). ESR: Excess stillbirth risk, i.e. [(OR-1)/OR] ×100%, where OR approximates the adjusted relative risk (Han women are the reference group).

^a^ Adjusted for the effects of maternal age, parity, and education level.

^b^ Adjusted for the effects of maternal age, parity, education level, smoking, BMI, height, occupation, economic stress, IUD use, folate use, adverse pregnancy history.

^c^ Adjusted for all the above plus preterm birth.

Supplementary Table C- Adjusted odds ratios for stillbirth of ethnic minorities excluding deliveries with congenital anomalies: Yunnan, China, 2010-2018

|  | Crude  OR | ESR  (%) | Model 1^a^ | | Model 2^b^ | | Model 3^c^ | |
| --- | --- | --- | --- | --- | --- | --- | --- | --- |
|  |  |  | Adjusted OR | ESR  (%) | Adjusted OR | ESR  (%) | Adjusted OR | ESR  (%) |
| Dai | 1.79 (1.41, 2.26) | 44.13 | 1.68(1.32, 2.14) | 40.48 | 1.61 (1.26, 2.05) | 37.89 | 1.25 (0.97, 1.61) | 20.00 |
| Hani | 1.27 (0.89, 1.79) | 21.26 | 1.17 (0.83, 1.66) | 14.53 | 1.10 (0.78, 1.57) | 9.09 | 0.91 (0.63, 1.31) | -9.89 |
| Miao | 0.98 (0.67, 1.43) | -2.04 | 0.85 (0.58, 1.25) | -17.65 | 0.88 (0.59, 1.30) | -13.64 | 0.75 (0.50, 1.13) | -33.33 |
| Yi | 1.20 (1.03, 1.41) | 16.67 | 1.15 (0.98, 1.36) | 13.04 | 1.12 (0.95, 1.32) | 10.71 | 1.21 (1.02, 1.43) | 17.36 |

*Note.* OR: Odds ratio (Han women are the reference group). ESR: Excess stillbirth risk, i.e. [(OR-1)/OR] ×100%, where OR approximates the adjusted relative risk (Han women are the reference group).

^a^ Adjusted for the effects of maternal age, parity, and education level.

^b^ Adjusted for the effects of maternal age, parity, education level, smoking, BMI, height, occupation, economic stress, IUD use, folate use, adverse pregnancy history.

^c^ Adjusted for all the above plus preterm birth.

Supplementary Table D- Adjusted odds ratios for stillbirth of ethnic minorities including deliveries with missing plurality and gestation age: Yunnan, China, 2010-2018

|  | Crude  OR | ESR  (%) | Model 1^a^ | | Model 2^b^ | | Model 3^c^ | |
| --- | --- | --- | --- | --- | --- | --- | --- | --- |
|  |  |  | Adjusted OR | ESR  (%) | Adjusted OR | ESR  (%) | Adjusted OR | ESR  (%) |
| Dai | 1.81 (1.46, 2.26) | 44.75 | 1.73 (1.38, 2.17) | 42.20 | 1.65 (1.31, 2.07) | 39.39 | 1.29 (0.97, 1.59) | 22.48 |
| Hani | 1.22 (0.88, 1.71) | 18.03 | 1.15 (0.82, 1.60) | 13.04 | 1.08 (0.78, 1.52) | 7.41 | 0.90 (0.64, 1.28) | -11.11 |
| Miao | 0.97 (0.67, 1.39) | -3.09 | 0.87 (0.60, 1.25) | -14.94 | 0.90 (0.62, 1.31) | -11.11 | 0.77 (0.53, 1.14) | -29.87 |
| Yi | 1.14 (0.98, 1.33) | 12.28 | 1.11 (0.95, 1.29) | 9.91 | 1.07 (0.92, 1.26) | 6.54 | 1.16 (0.98, 1.36) | 13.79 |

*Note.* OR: Odds ratio (Han women are the reference group). ESR: Excess stillbirth risk, i.e. [(OR-1)/OR] ×100%, where OR approximates the adjusted relative risk (Han women are the reference group).

^a^ Adjusted for the effects of maternal age, parity, and education level.

^b^ Adjusted for the effects of maternal age, parity, education level, smoking, BMI, height, occupation, economic stress, IUD use, folate use, adverse pregnancy history.

^c^ Adjusted for all the above plus preterm birth.
